# Scleral Cross-Linking Using Riboflavin UVA Irradiation for the Prevention of Myopia Progression in a Guinea Pig Model: Blocked Axial Extension and Altered Scleral Microstructure

**DOI:** 10.1371/journal.pone.0165792

**Published:** 2016-11-09

**Authors:** Shuai Liu, Shengjie Li, Bingjie Wang, Xiao Lin, Yi Wu, Hong Liu, Xiaomei Qu, Jinhui Dai, Xingtao Zhou, Hao Zhou

**Affiliations:** 1 Department of Ophthalmology & Visual Science, Eye & ENT Hospital, Shanghai Medical College, Fudan University, Shanghai, China; 2 The Department of Pre-hospital Emergency, the First People's Hospital of Hefei, Anhui, China; 3 Key Laboratory of Myopia, Ministry of Health, Fudan University, Shanghai, China; 4 Shanghai Key Laboratory of Visual Impairment and Restoration, Fudan University, Shanghai, China; 5 Department of Clinical Laboratory, Eye & ENT Hospital, Shanghai Medical College, Fudan University, Shanghai, China; 6 Infections department, the People's Hospital of Fuyang, Anhui Medical University, Anhui, China; National University of Ireland—Galway, IRELAND

## Abstract

**Purpose:**

To develop methods of collagen cross-linking (CXL) in the sclera for the treatment of progressive myopia and to investigate the biomechanical and histological changes that occur in as a result.

**Methods:**

Twenty 14-day-old guinea pigs were divided into 3 groups: the cross-linking group (CL, n = 8), non cross-linking group (NCL, n = 8), and control group (n = 4). The scleras of the right eyes of the guinea pigs in the CL group were surgically exposed and riboflavin was dropped onto the irradiation zone for 20 seconds prior to ultraviolet-A (UVA) irradiation. The same procedure was conducted on the NCL group but without UVA irradiation. No procedure was conducted on the control group. The right eyes of the guinea pigs in the CL and NCL groups were then fitted with -10.00DS optics for six weeks. Retinoscopy and the axial lengths (AXL) were measured at baseline, and at the second, fourth and sixth weeks post-treatment in all three groups. All animal subjects were euthanized after the sixth week and then biomechanical and histopathological examinations of the scleras were conducted.

**Results:**

The mean AXL of the NCL group was longer than both the control and CL groups at six weeks (P = 0.001). The mean refractive error in the NCL group was statistically significantly more negative than both the control and the CL groups at six weeks (P = 0.001). The scleral collagen fiber arrangements of the CL and control groups were denser and more regularly distributed than the NCL group. Ultimate stress of the sclera was lowest in the NCL group, followed by the CL then the control group (P<0.05). Ultimate strain (%) of the sclera was lowest in the CL group followed by the NCL and then the control group (P<0.05).

**Conclusion:**

Our study demonstrates that scleral CXL using riboflavin UVA irradiation effectively prevents the progression of myopia by increasing scleral biomechanical strength in a guinea pig model.

## Introduction

Vision is one of the most important sensory perceptions for an individual to connect with the world in which they live. With the increasing use of our eyes for near work in today’s modernized society, myopia is becoming more prevalent as a cause of reduced vision. The prevalence of myopia has been reported to be around 30% in the general population in the United States of America, but much higher prevalence rates of up to 60% have been reported in some Asian countries.[1] Fortunately, most myopic patients can have their refractive errors corrected by the use of optical or surgical corrections, in order to achieve good visual acuity. However, extremely high myopia carries substantial risks, as it can lead to many pathological changes in the eye, such as staphyloma and retinal detachment, which may result in permanently reduced vision and even blindness.[2]

There have been many factors that have been demonstrated to contribute to the development of myopia, including genetic factors, visual deprivation and prolonged near work.[3–5] Animal studies have also shown that long term defocus can lead to high myopia, however the exact pathophysiology of myopia development is still unclear. Therapeutic attempts to arrest myopic progression include optical correction and the administration of cycloplegic drops, such as atropine or tropicamide.[6, 7] Scleral strengthening surgery, such as polymeric gel injection or scleral reinforcement surgery,[8] where a donor sclera or silicone band is sutured onto the sclera at the back of the globe to prevent excessive eye globe extension, are highly invasive procedures that have been demonstrated to have limited success at halting myopic progression.[9] The sclera must first become biomechanically weaker before it can be stretched as a result of myopia. This is often caused by enzymes. Thus, it is also necessary to increase the resistance of the sclera against enzymatic digestion.[10]

Myopia is known to lead to structural changes in the eye. It leads to progressive scleral thinning, resulting in a decrease in collagen fiber circumference and disturbances of collagen fibrillogenesis and fibrolamellar reconstruction. These changes occur mainly at the equator of the sclera.[11, 12] It has been shown that impaired CXL is an important factor in the development of the myopic sclera.[13] The effects of treatments altering the strength of the cornea and sclera are manifested in changes in the rate and extent of deformation of the globe under the influence of intraocular pressure. Wollensak et al. were the first to propose cross-linking treatment of sclera for the prevention of myopic progression, similar to their cross-linking approach to stop the progression of keratoconus. They applied photosensitizer riboflavin combined with ultraviolet-A (UVA) irradiation to induce artificial scleral CXL, and found a 157% increase in the rigidity of porcine and human scleras in vitro.[14, 15]

UV-riboflavin cross-linking therapy is based on the principle that when the photosensitizer riboflavin undergoes ultraviolet wavelength irradiation, it becomes excited to a triplet state and generates reactive oxygen species that may induce a cross-linking reaction (photochemical reaction) between the various molecules and amino-induced collagen fibers, thereby increasing the mechanical strength of the sclera and its resistance to the expansion of collagen fibers as well as chemical resistance against collagenases.[14, 15]

To date, there are no studies in the literature investigating the biomechanical and microstructural changes induced in the sclera following CXL using riboflavin UVA irradiation for the treatment of progressive myopia. In 2014, Dotan *et al*. demonstrated the effect of this cross-linking treatment on myopic model rabbits, but they did not study the relationship between the biomechanical phenomenon and the microstructure of the sclera.[16] The present study aims to investigate the biomechanical and collagen fiber changes that occur in the sclera after cross-linking treatment, to provide evidence for a potential surgical approach to the treatment of progressive myopia.

## Methods

### Animals

Twenty 14-day-old guinea pigs weighing between 100g to 130g were used in this experiment. Guinea pigs of different sex were included randomly. All experiments were conducted in accordance with the ARVO Statement for the Use of Animals in Ophthalmic and Vision Research and the regulations of The Eye & ENT (EENT) Hospital affiliated with Fudan University (Shanghai, China) for animal experimentation. Guinea pigs were obtained from the laboratory of Fudan University, China. We confirm that Institutional Animal Care and Use Committee (IACUC) approved this study.

### Experiment procedure

The twenty guinea pigs were divided into three groups. We numbered the guinea pigs from 1 to 20, then used the calculator tool on a computer to generate random numbers for selection of the guinea pigs. Eight were randomly allocated to the cross-linking group (CL group); eight were randomly allocated to the non cross-linking group (NCL group); and the remaining four were allocated to the control group.

In all groups, only the right eye was used in the experiment, and nothing was performed on the fellow eye.

In the CL group, at baseline, the refractive error was measured with retinoscopy and AXL was measured with an A ultrasound. The guinea pig then underwent the CXL surgical procedure (as described in the next section). After the procedure, a -10.00D lens was placed in front of the surgical eye and the same measurements at baseline was repeated at the second, fourth and sixth week.

In the NCL group, the same experimental procedure as the CL group was conducted. With the only exception that UVA irradiation was not performed during the surgery procedure.

In the control group, no surgery or optical lenses were used on the guinea pigs. They were bred according to standard protocol. Refractive error and AXL measurements were performed at the same frequency as the experimental groups.

Six weeks later, all animal subjects were euthanized and their right eyes were enucleated. Biomechanical measurements and histopathological examinations of the scleral specimens were performed on the scleral specimens obtained.

### Defocus-induced myopia model

**Fig 1:**
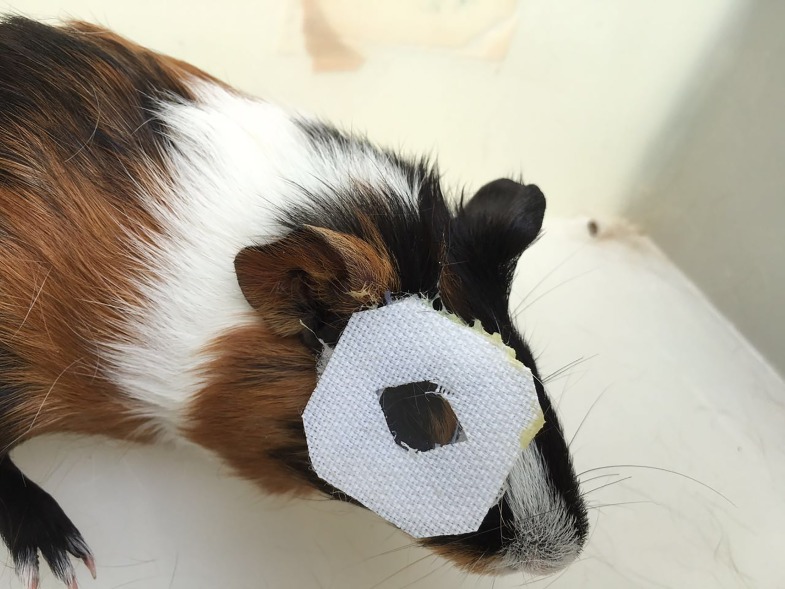
A guinea pig with the defocussing optic in place.

The right eyes of the guinea pigs in the CL and NCL groups wore -10.00DS optics after the CXL procedure was performed on the guinea pigs in both CL and NCL groups, (approximately 14 days after birth). The optics were purchased from the Optometry center at the EENT Hospital. The animals were first anesthetized with chloral hydrate, and the hair around their right eyes was removed with a pair of scissors. The optics was fixed onto the right eyes of the guinea pigs using double-sided tape (Fig 1). The double-sided tape was chosen to be thick enough such that a gap was left between the eye and the optic for easier cleaning of the optics and protection of the cornea.

### CXL surgery procedure

**Fig 2:**
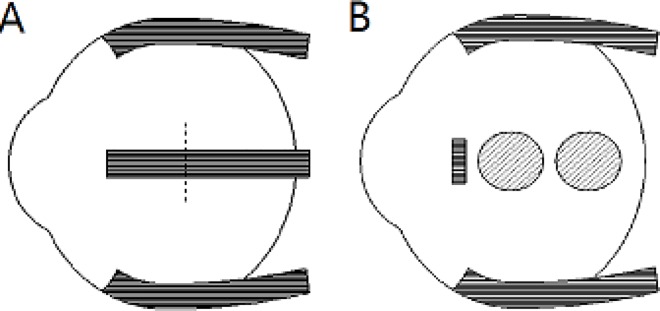
External view of muscle reflection (A). The lateral rectus was cut off in the position of the imaginary line. External view of the spot distribution of the two irradiation zonesper quadrantin each guinea pig (B).

At 14 days old, the CXL procedure was conducted on the right eye of all guinea pigs in the CL and NCL groups. They were anesthetized with chloral hydrate prior to undergoing the procedure. The guinea pigs underwent 360-degree conjunctival peritomy. The right eyeballs of the guinea pigs in the CL group were divided into two sides: the nasal side and the temporal side. Due to the small size of the guinea pig’s eyes, the medial and lateral recti were excised from the muscle insertion sites in order to explore the equatorial sclera. (Fig 2A), and were not reattached after the surgery. Each side had two irradiation zones, one at the equatorial sclera and one at the posterior sclera (Fig 2B). The area of each zone measured 0.13 cm^2^ with a radius of 2 mm. Photosensitizer solution containing 0.1% dextran-free riboflavin-5-phosphate was instilled as a drop onto each irradiation zone for 20 seconds before irradiation and then every 20 seconds during the 300-second irradiation period. In total, there were 4 irradiation sites per eye, (2 irradiation zones per side) with a total irradiation time of 1200 seconds per eye. The irradiation and the instillation of riboflavin were conducted simultaneously by the same two surgeons (Liu S and Li SJ). The irradiation device was an optical device which emitted an UVA (370 nm) light source. The UVA calculation was preset by the equipment, and the irradiation distance was kept constant at 2–3cm. The device was set to 57 mW/cm^2^ according to the method described elsewhere.[16] UVA light (370 nm) was applied perpendicular to the sclera, at 57 mW/cm^2^.

### AXL measurement

After the instillation of topical anesthesia (10% chloral hydrate), three measurements each were taken in the right eyes for the anterior chamber depth, AXL and lens thickness using an ultrasound A-scanner (Opticon, Italian, software version: Opticon 2000SPA, instrument accuracy of ±0.036 mm) and then averaged. A basic ocular examination including fundoscopy with a direct ophthalmoscope was also performed.

### Scleral specimens

The cross-linked right eyes were used for biomechanical measurements and histopathological examination. At the end of the experiment, the guinea pigs were sacrificed with an overdose of a solution containing ketamine (95 mg/ml) and xylazine (5 mg/ml) and the right eye globes were removed. Two strips of sclera from the surgical sites were removed (one each from the temporal and nasal sides). Each strip was 8x4mm in size. The temporal side of the sclera was used for biomechanical measurements, while half of the nasal side (4*4mm^2^) was used for electron microscopy and the other half (4*4mm^2^) was used for histopathological examination.

### Biomechanical measurements

Biomechanical measurements were performed on the 20 scleral specimens from the CL group (n = 8), NCL group (n = 8) and the control group (n = 4). Biomechanical properties of the sclera were tested on an Instron 5565 universal testing machine at 25±5°C and 50±5% relative humidity. For the tensile tests, the samples measured were 5 mm in length, 4 mm in width and approximately 0.2 mm thick. During testing, the gauge length was 2 mm and the cross-head speed was 10 mm/min.

### Histopathological examination

Histopathological examinations were performed on the 20 scleral specimens from the CL group (n = 8), NCL group (n = 8) and the control group (n = 4) using an electron microscope and an optical microscope. The specimens were fixed in 4% neutral buffered formalin for at least 1 week prior to light microscopy. 4 x 1um thin paraffin sections were sectioned from these samples and stained with hematoxylin–eosin, periodic acid-Schiff stain (PAS). The slides were evaluated using a Zeiss light microscope (Axiomat; Zeiss, Oberkochen, Germany) at 40–1000 X magnification. The specimens were fixed in 25ml/L glutaraldehyde solution for at least 24 hours prior to electron microscopy (scanning electron microscope). 1mm×1mm×1mm sections were cut and rinsed in phosphate buffer solution, followed by osmium tetroxide fixation, then dehydrated and embedded before observation with the electron microscope. The circumference of the collagenous fibers, the number of collagenous fibers and the percentage of collagenous fiber area from the electron microscope photographs of the sclera sections were evaluated with an image processing software (ImageJ 1.48v, Wayne Rasband, National institutes of health, USA). A threshold value of 100–800nm for the collagenous fiber circumference was set to ensure accuracy of the results.

The fibre bundle circumference was used as a surrogate measurement method of the size of the fibre bundles due to an inability of the imaging software to recognize and directly calculate the diameter of the fibre bundles.

### Statistical analysis

The data were analyzed using SPSS13.0 software (SPSS Inc., Chicago, IL). Results were presented as mean±SD. The non-parametric Kruskal-Wallis H test was used to compare differences within each of the three groups. The Mann-Whitney U test was used to compare difference between groups. A p value of <0.05 was considered statistically significant.

## Results

**Fig 3:**
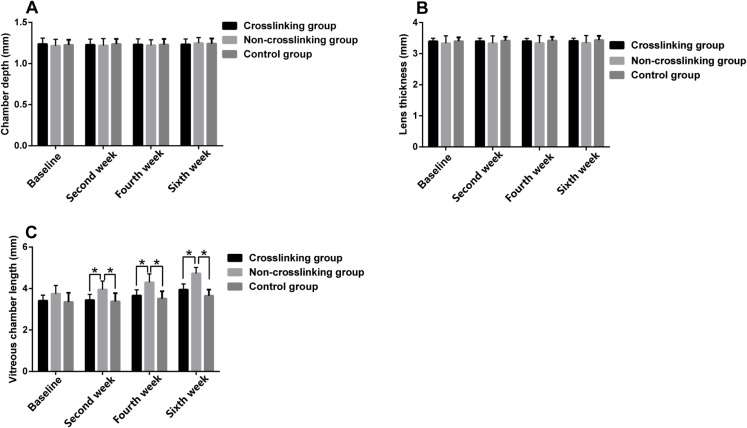
The comparison of anterior chamber depth (A), lens thickness (B), vitreous chamber length (C) among the three groups. Top of the box plot represents the mean and the bar of each box represents the standard deviation. *Statistical significance (p < 0.05) between the groups.

**Fig 4:**
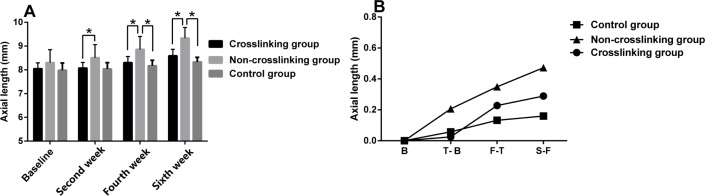
The comparison of axial length (A) between the three groups. Top of the box plot represents the mean and the bar of each box represents the standard deviation. The trend graph of axial length (B). Triangle, square and circle represent the means. *Statistical significance (p < 0.05) between the groups. B: the value of axial length (baseline); T-B: the value of axial length change (second week minus baseline); F-T: the value of axial length change (fourth week minus second week); S-F: the value of axial length change (sixth week minus fourth week).

**Fig 5:**
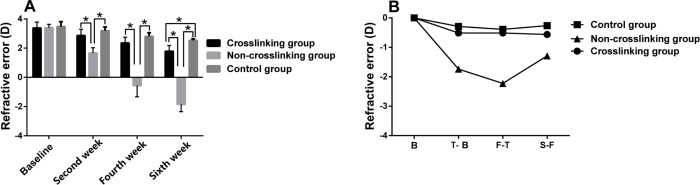
The comparison of refractive error (diopter) (A) between the three groups. Top of the box plot represents the mean and the bar of each box represents the standard deviation. The trend graph of refractive error (B). Triangle, square and circle represent the means. *Statistical significance (p < 0.05) between the groups. B: the value of refractive error (baseline) T-B: the value of diopter change (second week minus baseline); F-T: the value of diopter change (fourth week minus second week); S-F: the value of diopter change (sixth week minus fourth week).

Fig 3 and Table 1 represent the anterior chamber depth, lens thickness and vitreous chamber length measurements of the control, NCL and CL groups at the different measurement times. There were no statistically significant differences in the anterior chamber depth (p>0.05) or lens thickness (p>0.05) between the three groups at the different measurement times. In the NCL group, vitreous chamber length was significantly longer than the control group and the CL group at the second (P = 0.028), fourth (P = 0.005) and sixth (P = 0.001) weeks post-treatment. The AXL of the NCL group was longer than the CL group at the second (p = 0.048), fourth (p = 0.015) and sixth weeks (p = 0.002) post-treatment. The AXL of the NCL group was longer than the control group only at the fourth (p = 0.027) and sixth weeks (p = 0.004) post-treatment (Fig 4A). The mean change in AXL in the NCL group was higher than the control and CL groups as seen in Fig 4B. The refractive error in the NCL group was statistically significantly lower (more myopic) than the control group and the CL group at the second (P = 0.001), fourth (P = 0.001) and sixth weeks (P = 0.001) post-treatment (Fig 5A). The control and CL groups did not exhibit a statistically significant difference in refractive error at the second or fourth weeks, however by the sixth week, there was a significant difference in refractive error between the control and CL groups, with the CL group demonstrating a less positive refractive error (more myopic) than the control group (P = 0.001) (Fig 5A). The mean change in refractive error was greater in the NCL group than the control and CL groups, and the mean change in refractive error was similar between the control and CL groups (Fig 5B). There were no statistically significant differences (p>0.05) in the anterior chamber depth, lens thickness, refractive error, vitreous chamber length or AXL at the different measurement times between the control and CL groups, with the exception of the refractive error at the sixth week (P = 0.001).

**Table 1:** Comparison of anterior chamber depth, lens thickness and vitreous chamber length among the three groups.

	Crosslinking group		Non-crosslinking group		Control group			
	mean	SD	mean	SD	mean	SD	F	p
Pre-experiment								
ACD	1.24	0.07	1.22	0.08	1.23	0.06	0.240	0.887
LENS	3.40	0.10	3.34	0.24	3.40	0.12	1.112	0.573
AXL	8.05	0.24	8.30	0.54	7.98	0.03	0.829	0.661
Refractive error	3.38	0.41	3.40	0.23	3.48	0.33	0.416	0.812
VCL	3.41	0.27	3.75	0.40	3.35	0.44	3.023	0.221
Second week								
ACD	1.23	0.07	1.22	0.08	1.24	0.06	0.111	0.946
LENS	3.40	0.10	3.34	0.24	3.42	0.12	1.456	0.483
AXL	8.07	0.24	8.51	0.55	8.04	0.26	5.061	0.075^c^
Refractive error	2.86	0.44	1.66	0.37	3.19	0.27	14.565	0.001^b^^,^^c^
VCL	3.44	0.27	3.96	0.41	3.39	0.39	7.117	0.028^b^^,^^c^
Fourth week								
ACD	1.23	0.07	1.22	0.07	1.23	0.07	0.067	0.967
LENS	3.40	0.09	3.34	0.24	3.42	0.12	1.035	0.596
AXL	8.30	0.26	8.86	0.55	8.17	0.23	8.173	0.017^b^^,^^c^
Refractive error	2.35	0.40	-0.56	0.77	2.80	0.25	15.200	0.001^b^^,^^c^
VCL	3.67	0.27	4.30	0.41	3.52	0.35	10.582	0.005^b^^,^^c^
Sixth week								
ACD	1.23	0.07	1.25	0.07	1.24	0.062	0.381	0.826
LENS	3.41	0.09	3.35	0.23	3.44	0.13	1.604	0.448
AXL	8.59	0.28	9.33	0.45	8.33	0.19	13.125	0.001^b^^,^^c^
Refractive error	1.79	0.39	-1.85	0.49	2.54	0.11	16.494	<0.001^a^^,^^b^^,^^c^
VCL	3.95	0.28	4.73	0.28	3.65	0.30	14.786	0.001^b^^,^^c^

Data are expressed as mean±standard deviation (SD). ACD: anterior chamber depth. LENS: lens thickness. VCL: vitreous chamber length. AXL: axial length.

Kruskal-Wallis Hand Mann-Whitney U tests were used.

^a^P<0.05 for the difference between Crosslinking group and Control group.

^b^P<0.05 for the difference between Non-crosslinking group and Control group.

^c^P<0.05 for the difference between Crosslinking group and Non-crosslinking group.

**Fig 6:**
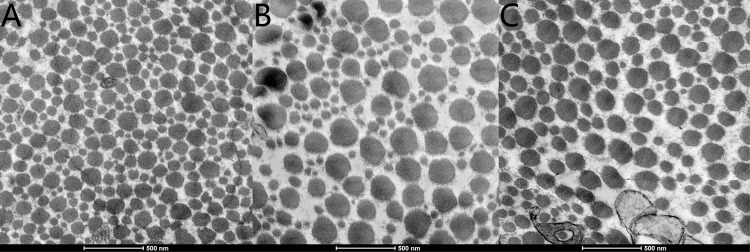
Electron microscope photographs illustrating collagenous fiber in control group (A), crosslinking group (B), and Non-crosslinking group (C). Cross sections taken from sclera. The scale can be found in the figure. The scleral collagen fiber of NCL group were scattered and irregularly distributed in C. The scleral collagen fibers of the CL group were more regularly distributed in B compared with C. The scleral collagen fibers of the control group were denser and regularly distributed in A compared with B.

**Fig 7:**
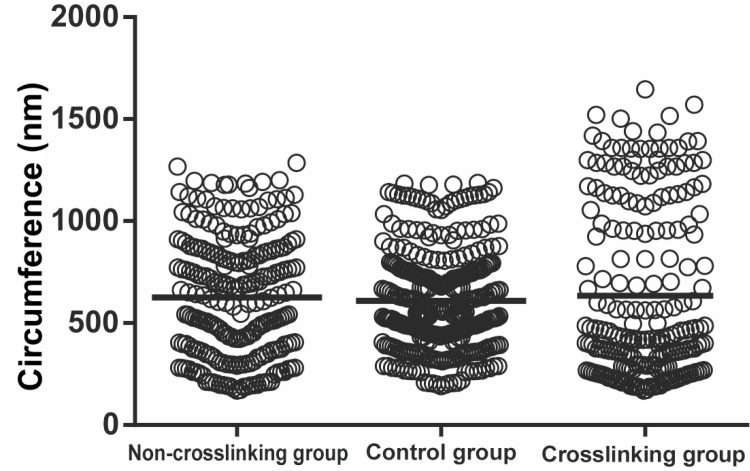
Comparison of the circumference of collagenous fibers among the three groups. Each data point represents one subject. Middle of the chart represents the mean.

**Fig 8:**
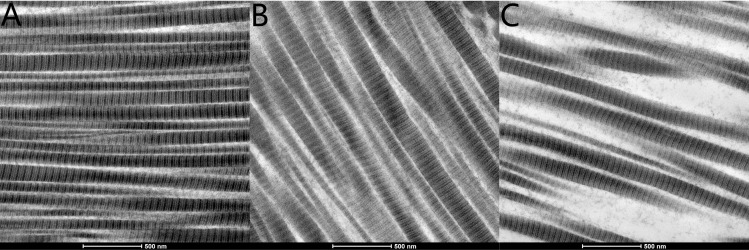
Electron microscope photographs illustrating collagenous fiber arrangements in the control group (A), CL group (B), and NCL group (C). Longitudinal sections taken from the sclera. The scale can be found in the figure. The scleral collagen fibers of the NCL group were loose and irregularly distributed in C. The scleral collagen fibers of the CL group were more dense and regularly distributed in B compared with C. The scleral collagen fibers of the control group were more dense and regularly distributed in A compared with B.

Histopathological evaluation of the CL, NCL and control groups using electron microscopy evaluated the circumference of collagenous fibers, the number of collagenous fibers and the percentage of collagenous fiber area (Figs 6, 7 and 8 and Table 2). The number of scleral collagen fibers and the percentage of collagenous fibres per unit area were higher in the control group than in the CL and NCL groups (F = 19.314, P<0.001 and F = 16.447, P<0.001 respectively) (Figs 6 and 7 and Table 2). There was no significant difference in the circumference of the collagenous fibers between the three groups (p>0.05). The distribution of the scleral collagen fibers in the NCL group was more scattered and irregular (Fig 7C), whereas in the CL group, the distribution of the scleral collagen fibers was more regular than the NCL group (Fig 7B). The scleral collagen fiber distribution of the control group was similar in appearance to the CL group (Fig 7A). Longitudinal sections of the scleral collagen fibers in the control and CL groups revealed a dense and regular distribution, whereas the NCL group showed a loose and irregular distribution (Fig 8).

**Table 2:** Histological Evaluation of Scleras By Electron Microscopy.

	Crosslinking group	Non-crosslinking group	Control group	F	p
Number of fibre bundles	219±8	236±10	309±9	19.314	<0.001^a^^,^^b^
Circumference (nm)	619.55±8.71	614.62±10.28	609.82±7.52	4.805	0.090
Area (%)	70.82±3.75	67.07±5.46	84.97±2.67	16.447	<0.001^a^^,^^b^

Data are expressed as mean±standard deviation (SD).

Kruskal-Wallis H test was used.

^a^P<0.05 for the difference between Crosslinking group and Control group.

^b^P<0.05 for the difference between Non-crosslinking and Control group.

**Fig 9:**
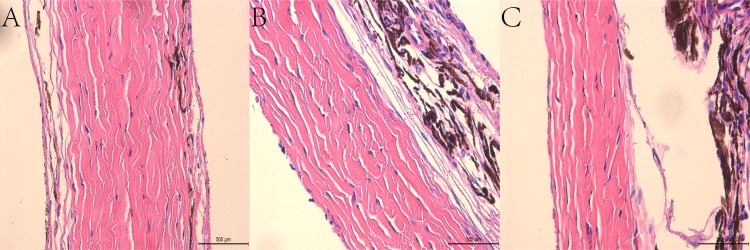
Light microscopy photographs illustrating scleras in the control group (A), CL group (B), and NCL group (C). (Haematoxylin and Eosin). Sections taken from the nasal side of the globe. The scale is presented in the figure. Collagenous tissue of the scleras in A were similar in appearance to B. Collagenous tissue of scleras in C were more loose and thinner in comparison to B.

Histopathological evaluation of the scleras in the CL and control groups using light microscopy showed no evidence of pathological findings, such as necrosis, inflammation, degeneration or atrophy. The sclera of the control group (1413.75±31.26 um) was thicker (t = 29.469, p<0.001) than the NCL group (765.25±37.77 um). The sclera of the CL group (1110.88±45.12 um) was also thicker (t = 16.615, p<0.001) than the NCL group (765.25±37.77 um). Collagenous tissue of the scleras in the CL group appeared dense and regular, similar in appearance to that of the control group. Therefore, no histological differences could be found between the scleral specimens in the CL and control groups (Fig 9); however, the collagenous tissue of the scleras from the NCL group appeared more loose and thin when compared with the CL and control groups (Fig 9). It should be noted that all scleral specimens of the eyes in the CL group examined using light microscopy were similar in appearance to those in the control group.

**Fig 10:**
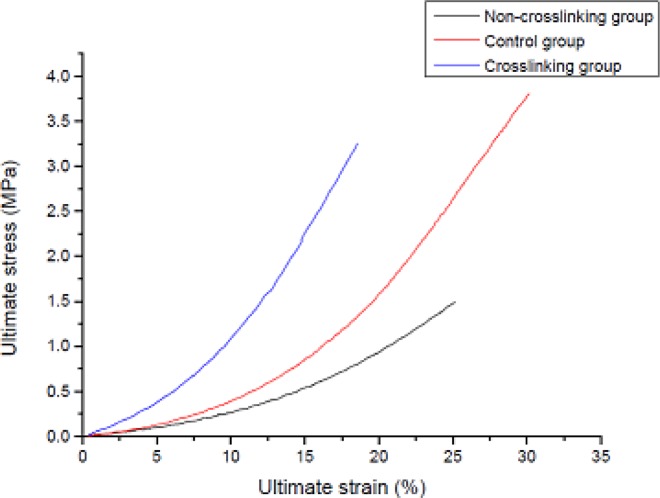
Stress-strain curve of the tensile test. Three curves were chosen randomly from each of the three groups in this study. Ultimate stress of the sclera was lowest in the NCL group followed by the CL group and the control group; Ultimate strain (%) of the sclera was lowest in the CL group followed by the NCL group and the control group.

Biomechanical measurements were performed on the 20 scleral specimens from the eyes in the CL group (n = 8), NCL group (n = 8) and control group (n = 4). There was a significant difference (p<0.05) in ultimate stress and ultimate strain (%) between the three groups (Table 3 and Fig 10). Ultimate stress of the sclera was lowest in the NCL group followed by the CL group then the control group, with significant differences observed between the three groups (P<0.001). Ultimate strain (%) of the sclera was lowest in the CL group followed by the NCL group and then the control group, with a significant difference also observed (P<0.001).

**Table 3:** The Results of Biomechanical Measurements Among The Three Groups.

	Crosslinking group	Non-crosslinking group	Control group	F	p
Ultimate stress (MPa)	3.25±0.04	1.54±0.07	3.79±0.19	16.457	<0.001^a^^,^^b^^,^^c^
Ultimate strain (%)	18.55±0.43	24.81±0.77	31.81±1.17	16.457	<0.001^a^^,^^b^^,^^c^

Data are expressed as mean±standard deviation (SD).

Kruskal-Wallis H test was used.

^a^P<0.05 for the difference between Crosslinking group and Control group.

^b^P<0.05 for the difference between Non-crosslinking group and Control group.

^c^P<0.05 for the difference between Crosslinking group and Non-crosslinking group.

## Discussion

The sclera is a dense, fibrous, viscoelastic connective tissue that forms the outer coat of the eye. It is now known that the sclera undergoes constant remodeling throughout life, and this remodeling can be dramatically altered by changes in the visual environment, producing changes in ocular size and the refractive state.[17] Scleral thinning and localized ectasia of the posterior sclera are characteristic changes of the myopic eye noted by many observers. This overall scleral thinning, observed in highly myopic human eyes, is associated with thinning of the collagen fiber bundles as well as a reduction in the size of the individual collagen fibrils, resulting in a preponderance of unusually small circumference fibrils averaging below 60–70 nm. Therefore, theoretically speaking, one proposed direct and effective method to control the development of high myopia, would be to increase the stiffness of the sclera to ultimately halt the thinning of the sclera, thereby halting myopic progression.

In the present study, the experimental guinea pigs were stimulated to develop myopia with the use of myopic lenses. The main anatomical change in myopia induced by optical defocus is the axial elongation of the vitreous body.[18, 19] In this study, the guinea pigs in the two experimental groups all experienced an elongation in vitreous body length when compared to the control group, indicating that the lens-induced defocus successfully induced myopia in these groups of guinea pigs. This result was in agreement with the findings of previous studies in myopic development.[18, 20] The guinea pigs in the two experimental groups also experienced an elongation in AXL as well as a negative shift of their refractive error. The success of the myopic modeling suggests that vitreous chamber elongation contributed the most to the overall axial elongation and the consequent myopic development.

In humans, the scleral tissue contains approximately 90% collagen by weight, which consists predominantly of type I collagen fibres.[21] The biomechanical scleral changes evident in myopia are due to a net loss of the collagen matrix and subsequent thinning of the sclera. This is evaluated by measuring the reduction in dry weight of the sclera, in addition to a decrease in the amount of type I collagen and an increase in the levels of both latent and active matrix metalloproteinase 2 (MMP-2; gelatinase-A).[17] The effect of cross-linking on the sclera has been studied through measurements of the mechanical strength of the scleral collagen in rabbit and human scleras. The same conclusion has been reached by a number of previous studies, who demonstrated that cross-linking can increase the firmness of the scleral tissue.[22, 23] One of the most plausible mechanisms of collagen cross-linking in CXL is thought to be the creation of additional chemical bonds between histidine, hydroxyproline, hydroxylysine, tyrosine, and threonine amino-acid residues.[24] Note that CXL has been reported to have a certain impact on the biochemical properties of corneal tissue by increasing the resistance of corneal matrix against digestion by proteolytic enzymes such as pepsin, trypsin, and collagenase.[10] The authors attribute the stabilizing biochemical effect of CXL to alterations in the tertiary structure of collagen fibrils, thus denying the proteolytic enzymes access to their target sites. Choi *et al*. conducted an in-vitro study using riboflavin and UVA for CXL, and reported a significant increase in the density and area of collagen fibrils following CXL compared to non-cross-linked human scleras.[23] Furthermore, they found an increase in the scleral thickness and an irregular parallel arrangement of collagen fibrils with increased circumferences.

Riboflavin UVA has been demonstrated to induce physical cross-linking of corneal collagen fibres and has been used successfully as a relatively strong cross-linking method for the treatment of progressive keratoconus.[14] Cross-linking of the sclera with rose bengal is ineffective due to poor scleral penetration,[25] therefore in the present study, rheomacrodex solution was used to dissolve the riboflavin, as the viscosity of rheomacrodex is strong enough to allow the dissolved riboflavin to adhere onto the sclera. We chose to irradiate the sclera at the scleral equator due to easy exposure, and also because in myopia, the changes in scleral structure occur mainly near the equatorial and posterior poles.[26] The location of the irradiation site was chosen to be as close to the posterior pole as possible without damaging the optic nerve. Through the excision of the medial and lateral recti muscles, the equatorial sclera can be adequately exposed, and the cornea is protected by the palpebral conjunctiva of the lower eyelid while the surgery is performed. During the procedure, riboflavin UVA appears yellow on the background of the whitish sclera; therefore the cross linking position can be visualized directly on the sclera, allowing for a more accurate localization of the cross linking position.

The present study found that both axial elongation and the development of myopia were halted in the CL group when compared to the NCL group. The biomechanical data showed that the scleras in the CL group were much stronger than those in the NCL group. CXL of the sclera was effective in increasing the ultimate strain of the sclera, making it stiffer and less resistant to stretching. The biomechanical indices reflect the tensile properties of the unit cross-sectional area of the specimen, which is independent of the thickness of the test piece; therefore differences in scleral thickness should not impact these measurements in the present study.

Furthermore, with the use of the electron microscope, we discovered a difference in the appearance of the fibre bundles between the three groups. The number of collagenous fibre bundles per unit area and the percentage of collagenous fibres per unit area were both reduced in the CL and NCL groups, although the CL group showed a denser and more regular distribution of their fibre bundles in comparison to the NCL group. This suggests that CXL of the sclera was able to maintain, to some extent, the microstructural organisation of the sclera in this myopic guinea pig model. However, despite the average circumference of the fibre bundles being similar between the three groups, the distribution of the sizes of the fibre bundles were distinctly different between the three groups. The CL group displayed a greater polarization in the sizes of the smaller and larger circumferences. As demonstrated in Fig 6B, where a greater difference in sizes between the small and large fibrils in the CL group resulted in a greater distribution of the data when plotted in Fig 7. The control group fibre bundles, on the other hand, were more regular in size, hence the distribution was closer towards the average (horizontal bar). The exact mechanism for polarization in the size of the fibre bundles in the CL group is unknown. Furthermore, the number of fibre bundles per unit area was lowest in the CL group when compared to the two other groups. This suggests that the increase in tissue stiffness and the enhanced ultimate stress values in the CL group, might be due to the formation of thickened collagen fibre bundles, and not hyperplasia of the sclera tissue.

In the NCL group, the collagen fibre bundles were not as dense as those in the control group. This decrease in the percentage area of fiber bundles might be due to overall axial elongation, which resulted in the stretching of the sclera, and thus leading to a reduction in the number of fiber bundles per unit area of sclera. Furthermore, the physical stretching of the fibre bundles might also make them appear thinner than the normal control group, which has been reported in previous studies.[27, 28] This might also explain why under light microscopy, the scleral thickness of the NCL group was much thinner in appearance than the CL group.

The direct impact of UV irradiation will depend on the wavelength, intensity and duration of irradiation. The present study irradiated equatorial scleral tissue using UV radiation, however, any collateral damage to the cornea and endothelial cells may be quite minimal. The light microscopy images showed no cellular oedema, deformation or melting, no withered nuclear or disintegrated perinuclear vacuoles, and no collagen fiber edemaor fracture. Fig 8 shows mitochondrial edema of fibroblast cells, however we believe these changes are common in acute phase reactions and are a part of the normal self-healing process.

In conclusion, we present in this study a guinea pig myopic model and scleral tissue cross linking using riboflavin and UVA irradiation. This study found that cross-linking using UVA irradiation was able to enhance scleral firmness in a group of myopic guinea pigs, and thus successfully reduced myopia progression when compared to the group of NCL guinea pigs. Further experimental studies are needed to investigate the short and long term biomechanical effect of crosslinking for the prevention of progressive myopia.
